# Ensuring a safe and adequate blood supply during the COVID-19 pandemic: the Moroccan National Blood Center experience

**DOI:** 10.11604/pamj.2020.37.275.26639

**Published:** 2020-11-25

**Authors:** Sabah Bouhou, Mohammed Benajiba

**Affiliations:** 1 National Center for Blood Transfusion and Hematology, Rabat, Morocco

**Keywords:** Management, COVID-19, pandemic, blood transfusion centers

## Abstract

At the end of 2019, a new coronavirus was identified in people living in the city of Wuhan in China. Since the appearance of the first cases in Wuhan, the SARS-CoV-2 epidemic has spread to the international scale. The COVID-19 pandemic is considered by international scientific societies to have an impact on blood donation and transfusion activities. Efforts must be made at the level of the transfusion centres to ensure proper management of the impact of this health crisis on the availability and the safety of blood products. The National Blood Centre of Morocco (MNBC) has demonstrated a great reactivity and a great adaptability since the beginning of the epidemic and has implemented several measures to face this health crisis. These measures have been updated taking into account the updating of data from national health authorities and international scientific societies concerning this new virus.

## Commentary

The current SARS-CoV-2 pandemic is considered to have the potential to decrease and jeopardize the supply of blood products [1]. Thus, blood transfusion services must be ready to adapt quickly, in response to changing circumstances and when the supply of blood products is likely to be affected [1,2]. Indeed, collection restriction measures are likely to disrupt supplies and generate a shortage of blood products which is extremely detrimental in terms of patient care and the quality and safety of care. Maintaining blood collections is essential to ensure national self-sufficiency in blood products [3]. For the risk of transmission of SARS-CoV-2 through blood and its components, it is not well documented but it is likely to be a minimal theoretical risk [3,4]. The transfusion centres must ensure availability and strengthen the safety of blood products during this period [3,4].

The National Blood Centre of Morocco (MNBC) constitutes a scientific reference on a national scale. It is responsible for the implementation of the Ministry of Health's policy on blood transfusion and is responsible for carrying out several missions including scientific and epidemiological monitoring and technical support for Moroccan Regional Blood Centres (MRBC). Since the outbreak of the new coronavirus SARS-CoV-2 on 31^st^ December 2019 in China, the MNBC organized several brainstorming meetings in the presence of national experts in blood transfusion in order to provide information about the real risk of this virus in blood donors and in transfused patients. The MNBC was also looking for information on the impact that this health crisis could have on the continuity of the various blood transfusion activities.

The first data published on January 2020 by the international societies such as the European Centre of Disease Control (ECDC) and the American Association of Blood Banks (AABB) stipulated that donor selection is an essential element of blood safety during this new epidemic [5,6]. The first recommendations of these societies concerned the criteria of medical selection of blood donors. Based on this data, on 29^th^ January 2020, the MNBC implemented the first measures and started the exclusion of at-risk donors from the new coronavirus. He informed the doctors responsible for the medical selection of donors on the current epidemiological situation of the new coronavirus and the need to deepen the medical interview by looking for signs such as cough, fever, runny nose, diarrhoea or vomiting. The temperature of donors was taken at any suspicion of fever in a blood donor and the temporary deferral for a period of 28 days was started for anyone who has stayed in a country considered to be at risk on this virus or who has been in contact with a subject returning from a country at risk or who has presented recent respiratory symptoms of a viral nature. The donors have been informed for the need to take all the necessary preventive measures with regard to infection with this virus. The doctors of the medical selection in MRBC have been regularly informed about the update of the definition of the suspect case COVID-19 issued by the Moroccan health authorities.

Another axis on which the MNBC had worked is the safety of blood products. The World Health Organization (WHO) reported on 28^th^ February 2020 that the risk mitigation strategy with regard to SARS-CoV-2 may also include quarantine of plasma for transfusion as a safety measure [1]. On 14^th^ March 2020, the High Council of Public health (HCPH) stipulate that fresh frozen plasma that does not benefit from the pathogen mitigation technique is secured by quarantine [2]. As a precaution, on 23^rd^ March 2020, the MNBC established the quarantining of all plasma produced by the MRBC from 1^st^ February 2020 and not using it as therapeutic plasma. A national quarantine procedure has been drawn up by the national quality assurance service of the MNBC and sent to all MRBC. From 5^th^ May 2020, the MNBC updated monthly this decision based on the scientific data available to date in relation with this new coronavirus and that reported the absence of any case of transmission of SARS-CoV-2 by blood products [7,8].

In the same way, the potential for a viremic phase of SARS-CoV-2 is currently not known and is likely to be limited to severe disease, analogous to other respiratory infections. With the current knowledge gaps there are too many uncertainties to recommend testing of blood donors for SARS-CoV-2 at present [1,2]. The testing of the blood supply is premature in the absence of cases of transfusion transmission or demonstrated infectivity of the COVID-19 virus in blood collected from asymptomatic persons [7,8]. This type of measure has never been mentioned in transfusion during other epidemics with respiratory viruses (pandemic influenza, SARS-CoV, MERS-CoV etc.) or even with hemorrhagic fever virus [3]. Until the writing of this paper and based on all available scientific data, the MNBC did not introduce any SARS-CoV-2 screening technique in Moroccan blood establishments.

But although that the risk of SARS-CoV-2 blood transmission has not established, an haemo-vigilance system should be in place for helping to understand the risk from blood and components and the overall effectiveness of the measures taken by the blood service [3,8]. The blood service must ensure the reinforcement of post-donation information according to regulatory procedures [2,3,8]. The MNBC established from 1^st^ April 2020 the following measurements: the strengthening of the self-exclusion system for blood donors by adopting the new possible case definitions of COVID-19 from the Ministry of Health, the reinforcement of pre-donation interrogation and in particular the medical selection of donors by adopting the case definition of COVID-19 according to the updated recommendations of the Ministry of Health, the reinforcement of the post-donation information with particular attention during the 28 days following the donation, where the donor is required to inform the transfusion centre of any evocative sign that could suspect an infection with SARS-CoV-2, the providing donors with the COVID-19 self-exclusion form prepared by the CNTSH so that donors can provide feedback through the contact means available to them, namely telephone numbers and address electronic, (donors can be contacted at regular intervals after the donation for information to this effect), the reinforcement of the haemo-vigilance system at MRBC level and collaboration with care services for the establishment of traceability of blood products transfused during this critical period and for the collection of any transfusion incident whatever its nature.

Donor and staff safety is another priority for the MNBC. Protective measures have been established since the start of the SARS-CoV-2 epidemic and reinforced after the declaration of the first case of SARS-CoV-2 in Morocco on 2^nd^ March 2020. These measures concerned the providing blood donors and staff with appropriate protective measures (masks, gowns, gloves, shoes, antiseptic solutions, hydro alcoholic gel...), the incitement to compliance with barrier gestures and standard hygiene rules, the respect for the distancing measures recommended by the Ministry of Health and the possibility of making an appointment before blood donation to minimize the risk of donors gathering and condensing at the fixed centres sites. In the same context, the regional blood center had to program donations by group of 5 donors and to arrange the reception areas in order to avoid any gathering in the medical selection process or while waiting for the sample. For the administrative activities, the MNBC has established the reduction of the number of meetings to urgent meetings and meetings for monitoring urgent and priority actions, the reduction of the number of meeting participants and the setting up a meeting by videoconference as many opportunities as possible. All missions involving travel between cities have been postponed except for urgent actions. At the level of laboratories activities, on 26^th^ March 2020, the MNBC established the monitoring of standard bio-security practices and a well-identified circuit of samples from suspect or infected COVID-19 patients received by MRBC as part of a request for LBP for blood transfusion. The activities at the level of donation biological qualification laboratories was organized so as to ensure continuity of work in this critical process even in the event of contamination of laboratory staff. The MNBC considered that the activity at the level of the biological qualification laboratories as a critical activity and that it must be maintained in order to continue to meet the needs in LBP. For this reason, since the beginning of the epidemic, the MNBC has continued to supply the MRBC with all equipment, reagents and fungibles necessary for the continuity and smooth running of activities at the transfusion centre level.

Since the declaration of the first case of COVID-19 infection in Morocco, in order to mitigate the impact of the COVID-19 pandemic on the reduction of availability of blood donors and labile blood products, the MNBC appointed a committee how was in charge of twice-daily monitoring of the national LBP stock and the daily collection of the number of blood donations made at each blood transfusion center. This committee was charged also to ensure the regulation between MRBC to face the event of a particular need expressed by a regional blood center for LBP. The deficit centres are supplied from other blood transfusion centers and the necessary logistics are provided by the MNBC. Unfortunately, after the declaration of the first case on COVID-19 in Morocco on 2^nd^ March, the number of donors has started to decrease and particularly after the introduction of restrictive measures concerning displacement issued by the Moroccan authorities from 19^th^ March. In March, the number of donations made nationally was 27,812, in April it was 22,415 donations and in May 17,147 donations. An emergency plan was launched at the MNBC to resolve this situation. This plan was based on these measures: encouraging the competent authorities to issue special authorizations for blood donors to go to blood centres, the establishment of a blood donation service at all blood transfusion centers from 9 am to 7 pm to recruit as many donors as possible, encouraging associations working in the field of blood donation to provide assistance to blood transfusion centres, in particular awareness-raising and transportation of donors from their homes to transfusion centres and from blood centres to their homes after giving blood, the coordination and organization of several campaigns for blood donation in partnership with several institutions: the Moroccan general directorate of national security, the Moroccan auxiliary forces, the Moroccan general directorate of territorial surveillance, the Moroccan customs department, the royal gendarmerie, the Moroccan Ministry of the Interior and others Moroccan institutions.

These actions have led to an increase in the number of donors with 25,157 blood donations nationally in June. This has had a positive impact on the national stock in LBP and has made it possible to satisfied the demands for blood, in particular the urgent demands and the demands of chronic multiple transfusion patients. Besides these actions the MNBC recommends, from April 2020, the strengthening of dialogue and collaboration between MRBC and clinical services because the implication of the care services is essential for the good management of requests for blood products particularly for clinical situations requiring urgent transfusions of LBP and for poly-transfused patients. In health emergencies and during epidemics, the management of blood requests at the level of transfusion centres and at the level of care services must be based on the concept of patient blood management [3,8].

The strategy of communication is very important in the COVID-19 pandemic. Blood service must communicate clearly to ensure that the national emergency response team, donors and recipients and the public are properly informed and understand planned actions [3,8]. The communication strategy adopted by the MNBC during this health crisis is part of a quality approach based on the satisfaction of internal and external customers. So since 29^th^ January 2020, the MNBC has continued to regularly inform the Moroccan health authorities of all the measures taken by the MNBC in the field of blood transfusion and has updated the data transmitted to the MRBC about the new coronavirus. He has established an on-call list at the national level to respond to any request for information and to resolve any problem related to the availability of stable and labile blood products. The MNBC and MRBC had, also, established continuous communication with healthcare services. Donors have been informed about the availability of phone numbers for information on donating blood and making appointments. The MNBC responded to all requests for information from the media and newspapers regarding blood donation.

To take part in a process of therapeutic management of the COVID-19 infected patient, the MNBC had established a clinical trial protocol on the use of COVID-19 convalescent plasma in patients infected with SARS-CoV-2 in Morocco. According to current scientific publications, plasma treatment of convalescent COVID-19 has proven to be of great benefit in the management of patients infected with SARS-CoV-2 [9,10]. It´s effectiveness concerned the improvement of clinical and radiological signs, the decrease in viral load, the stopping the use of invasive and non-invasive mechanical support after administration and an earlier hospital discharge rate compared to those who have not been transfused with convalescent plasma [9,10]. The Moroccan protocol was based on WHO and European Commission recommendations and has been submitted to the Moroccan Ministry of Health for approval.

For the same therapeutic purpose, the MNBC expressed a predisposition to ensure the supply for the COVID-19 infected patients in case of need for stable blood products such immune-globulins and albumin. For COVID-19 patients´ transfusion care, Moroccan blood transfusion centres have been required to prepare and deliver blood products to some hospitalized COVID-19 confirmed patients. The transfusion centres have been required to establish a well-identified circuit of samples from COVID-19 patients suspected or confirmed in compliance with bio security measures at the level of the laboratories of the transfusion centres.

Finally, it should be emphasized that through a strategy of listening, sharing and active communication, the staff of the various Moroccan transfusion centers demonstrated exceptional dedication and incomparable commitment during this difficult period while demonstrating a positive reactivity to the recommendations established by the MNBC.

Several recommendations have been established by the MNBC to ensure effective support for transfusion centres and to ensure the availability and safety of blood products. These recommendations, of course, are subject to adjustment based on changes in current conditions.

## References

[1] WHO Blood Regulators Network (BRN) (2020). Interim position paper on blood regulatory response to the evolving outbreak of the 2019 novel coronavirus SARS-CoV-2. WHO.

[2] (2020). Haut conseil de la santé publique, Complémentaire à l'avis des 7 et 24 février 2020 relatif aux mesures de prévention à appliquer aux donneurs de sang, produits sanguins labiles, cellules, tissus et organes ayant séjourné en zone à risque de transmission du virus SARS-CoV-2.

[3] World Health Organisation (2020). Maintaining a safe and adequate blood supply during the pandemic outbreak of coronavirus disease (COVID-19): interim guidance. WHO.

[4] Chang L, Yan Y, Wang L (2020). Coronavirus disease 2019: coronaviruses and blood safety. Transfus Med Rev.

[5] European Centre for Disease Prevention and Control (2020). Risk assessment: outbreak of acute respiratory syndrome associated with a novel coronavirus, Wuhan, China; First Update. ECDC.

[6] AABB's Transfusion Transmitted Diseases Committee (2020). Update: impact of 2019 novel coronavirus and blood safety.

[7] European Centre for Disease Prevention and Control (2020). Coronavirus disease 2019 (COVID-19) and supply of substances of human origin in the EU/EEA - First Update. ECDC.

[8] WHO (2020). WHO: guidance on maintaining a safe and adequate blood supply during the coronavirus disease 2019 (COVID-19) pandemic and on the collection of COVID-19 convalescent plasma: interim guidance, 10 July 2020. World Health Organization.

[9] Casadevall A, Pirofski LA (2020). The convalescent sera option for containing COVID-19. J Clin Invest.

[10] Zhang B, Liu S, Tan T, Huang W, Dong Y, Chen L (2020). Treatment with convalescent plasma for critically ill patients with severe acute respiratory syndrome coronavirus 2 infection. Chest.

